# Minimization of Temperature Ranges between the Top and Bottom of an Air Flow Controlling Device through Hybrid Control in a Plant Factory

**DOI:** 10.1155/2014/801590

**Published:** 2014-06-11

**Authors:** Seung-Mi Moon, Sook-Youn Kwon, Jae-Hyun Lim

**Affiliations:** ^1^Department of Multimedia Engineering, Kongju National University, 275 Budae-dong, Seobuk-gu, Cheonan-si, Chungcheongnam-do 331-717, Republic of Korea; ^2^Green Energy Technology Research Center, Kongju National University, 275 Budae-dong, Seobuk-gu, Cheonan-si, Chungcheongnam-do 331-717, Republic of Korea; ^3^Department of Computer Science and Engineering, Kongju National University, 275 Budae-dong, Seobuk-gu, Cheonan-si, Chungcheongnam-do 331-717, Republic of Korea

## Abstract

To maintain the production timing, productivity, and product quality of plant factories, it is necessary to keep the growth environment uniform. A vertical multistage type of plant factory involves different levels of growing trays, which results in the problem of difference in temperature among vertically different locations. To address it, it is necessary to install air flow devices such as air flow fan and cooling/heating device at the proper locations in order to facilitate air circulation in the facility as well as develop a controlling technology for efficient operation. Accordingly, this study compares the temperature and air distribution within the space of a vertical multistage closed-type plant factory by controlling cooling/heating devices and air flow fans harmoniously by means of the specially designed testbed. The experiment results indicate that in the hybrid control of cooling and heating devices and air flow fans, the difference in temperature decreased by as much as 78.9% compared to that when only cooling and heating devices were operated; the air distribution was improved by as much as 63.4%.

## 1. Introduction

As local communities are urbanized and such issues as reduction of growing area and productivity due to climate changes and food stability come to the fore, the practical use of plant factories is drawing interest with reflection of consumers' demands for the stable supply of high-quality farm products [1–6]. A plant factory is a system to produce plants around the year systematically regardless of the season by artificially forming a growing environment in a controlled facility [4]. To control the production timing and productivity and maintain uniform-quality of crops in such a plant factory, an advanced level of environmental control is essential for the appropriate growing conditions [7, 8].

Environmental elements that directly affect photosynthesis include light, temperature, humidity, CO_2_ concentration, and nutrient solution, all of which are also major factors that decide growing rates. To produce high-quality crops systematically, the proper growing condition for each type of crop should be kept uniform in the space [9–13]. Temperature distribution, for instance, is one of the most important environmental factors to keep crop growth constant [14–19]. If the growing area is large, the air flow is likely to be nonuniform with lower wind velocity, which causes temperature difference among cultivation beds in the place as well as difficulties in producing uniform-quality crops [20, 21]. In the case of vertical multistage form plant factories, such as vertical farm whose growing efficiency per unit area is outstanding, the scale of space is large and the heights of cultivation bed are different. Hence, the temperature distribution varies depending on the vertical location; the productivity and quality deteriorate accordingly, and the energy consumption increases as the growing period is extended. To maintain the proper wind velocity and uniform temperature distribution in the growing space, the proper capacity and location of an air-flow fan need to be determined with consideration of the spatial characteristics, and technologies to control these elements efficiently also should be adopted [20–32].

To this end, this study comparatively analyzes the temperature and air distribution in the space under the hybrid control of the air conditioning unit and air-flow fan with utilization of a vertical multistage form-closed plant factory. The experiment proves that the hybrid control method of the air conditioning unit and air-flow fan facilitates the air circulation in the growing space and effectively minimizes temperature difference of the cultivation bed located at the upper and lower sides.

This study consists of the following sections.  Section 2 examines a vertical multistage type of closed-type plant factory testbed, experimental environments, and control conditions.  Section 3 comparatively analyzes the actual measurements of temperature and air flow distribution with reflection of the four control conditions. Lastly, Section 4 presents the conclusion and direction of future study.

## 2. Experiment Environment

**Figure 1:**
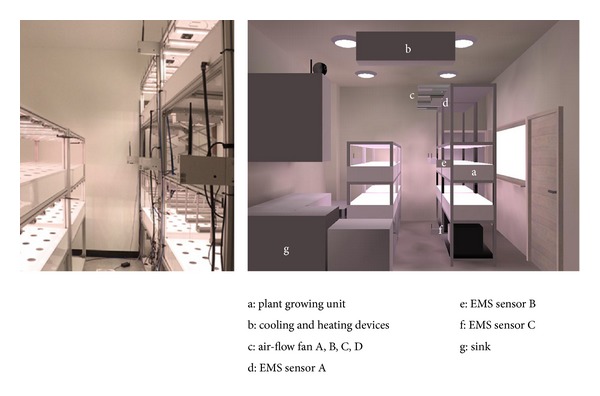
Experimental environment and facility.

This study utilized as a testbed the closed-type plant factory installed in a basement as large as 3, 470 mm × 4, 350 mm × 2, 970 mm (see Figure 1). The experiment space included such units as 4 sets of 2 columns, 1 row vertical type plant cultivation device, cooling and heating devices (S-W115AAW, 1030 mm × 325 mm × 250 mm, 37.5 m^2^) (b), 4 sets of air-flow fans for air circulation (SIV-200BC, 200 mm × 200 mm × 105 mm) (c), 3 sets (d–f) of integrated sensors to collect environmental information such as temperature, humidity, and CO_2_ real time, and sink (g) for growing tray cleaning and goods storage. In particular, the plant cultivation devices included the 2 height-variable sets of 1,315 mm × 2,470 mm × 605 mm and 2 fixed sets of 1,315 mm × 1,635 mm × 605 mm. At the top of each growing tray were 6 sets of bar type LED lightings, each of which included light sources effective for plant photosynthesis, which were red (630 nm, 660 nm), blue (430 nm, 450 nm), and white at the ratio of 11 : 4 : 3.

The hardware characteristics of cooling and heating devices and air-flow fans used in this experiment are presented in Table 1. The cooling and heating devices were fixed at about 45° on the wall at a side, and the temperature in the facility was set to 23°C with consideration of the growing condition of leaf vegetables. The air-flow fans were located in the middle of the plant cultivation unit with the supporter 2,370 mm from the ground.

The 3 sets of integrated sensors installed to monitor the environmental information within the facility could recognize the range of temperature from −40°C to +100°C. To check the errors among the collected data, the actual measurement test was conducted for about 1 hour at the same position. As a result, sensors B and C showed an error rate less than ±1% while the temperature measurement at sensor A was about 1°C lower than that at other sensors. Hence, the actual measurement at sensor A was analyzed after it was corrected in reference to the error rate. After the error rate test, 3 sets of the integrated sensors for environmental monitoring were installed 2,370 mm, 1,400 mm, and 70 mm from the ground, respectively, in order to measure the temperature at each height. Afterwards, the 3-dimensional WA-790 supersonic wave anemometer was utilized to measure the air distribution within the space, which would change depending on the controlling condition. The adopted 3-dimensional supersonic wave anemometer features the accuracy of ±(2% + 0.02 m/s) and the time division transmission changeover type supersonic wave pulse measurement. It measured 10 times per second over the range of 0–10 m/s.

**Figure 2:**
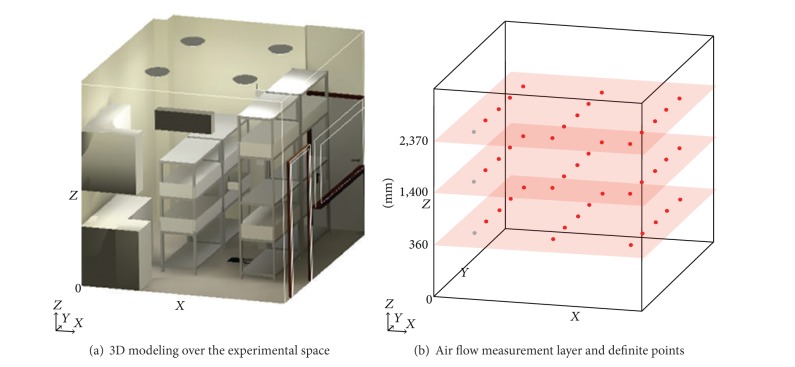
Standards for air flow distribution measurement.

Figure 2 shows the 3D modeling (a), measurement layers, and definite points (b) of the experiment space designed to actually measure the air flow distribution within the plant factory. Each layer in Figure 2(b) was located 360 mm, 1,400 mm, and 2,370 mm from the ground. 15 points of intersection in the grid were selected for actual measurement. The *XY* coordinate (0,0) of each grid was excluded from the actual measurement since a sink was located there.

## 3. Experiment and Analysis

This experiment aims to measure and analyze the temperature and air flow distribution at each point within the plant factory that would be changed by the hybrid control method of the air flow device. The controlling conditions depending on the operation settings of the air flow device are presented in Table 2. Among the controlling conditions, Cases 1 and 2, which did not consider the operation of cooling and heating devices, could not maintain the temperature setting of 23°C, and thus it was impossible to compare them directly with Cases 3 and 4. An experiment condition was added to grasp the effect of air-flow fans on minimizing difference in temperature among positions when no other units but LED lightings were operating in the plant factory which was sealed up. In addition, in the conditions of Cases 3 and 4, only the cooling and heating devices were operating exclusively or with air-flow fans. With the initial temperature set to 18.5°C, the experiment was conducted in different controlling conditions for 12 hours in total. In application of the graph library based on the PHP and HTML5 Canvas, the analysis module was designed with such functions as visualization of actual measurements and statistical analysis.

Prior to the experiment, the operation of all devices such as LED, air-flow fan, and so forth was stopped to check the air distribution in the space of a closed-type plant factory underground, and then the temperature was actually measured by the sensors as shown in Figure 1 for 12 hours. As a result, the average temperature at sensor A located on the top (2,970 mm) was 16.2°C, sensor B in the middle (1,400 mm) was 15.8°C, and sensor C at the bottom (70 mm) was 14.7°C. The difference between the top and bottom was 1.5°C on average. The difference in average temperature in the space between the initial point and ending point 12 hours after the experiment began was within 0.2°C, which indicated that changes in temperature outside did not affect the experiment space significantly because of physical characteristics. The scale of the plant factory was relatively small and it was located in a basement that was sealed up.

**Figure 3:**
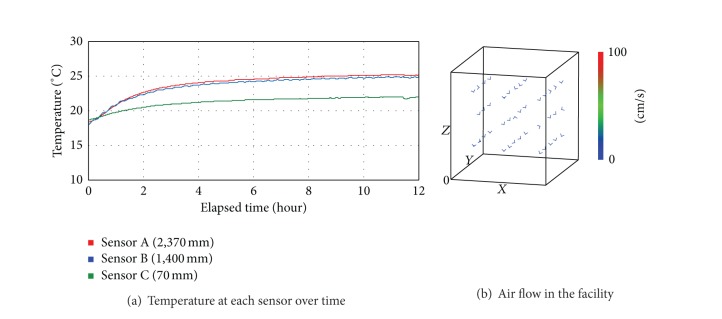
Temperature and air flow in the condition of Case 1.

Figure 3 shows the actual measurements of the temperature and air flow in the controlling condition of Case 1 as shown in Table 2. In the temperature Figure 3(a), the average temperature from the point of turning LED lightings on continued to increase from 18.4°C to 24.0°C. In comparison to the temperature at the top and at the bottom when LED lightings were turned on and 12 hours from that point, the temperature increased from 0.3°C to 3.2°C, which indicated that as the running hours of lightings increased, the difference in temperature increased accordingly. As for the average temperature at each sensor for 12 hours, that of sensor A on the top was 23.9°C, that of sensor B in the middle was 23.6°C, and that of sensor C at the bottom was 21.2°C, which meant that the difference in temperature between the top and bottom was about 2.7°C.  Figure 3(b) shows the air flow distribution at each position inside, and the colors and lengths of the arrows indicate the velocity at each measuring point. With the controlling condition of Case 1 applied, the average air flow rate was 0.057 m/s, and there was little change in air flow. In the experiment condition where air circulation was not smooth, plant growth and product quality might be adversely affected, and thus it was necessary to add and operate more air flow devices accordingly.

**Figure 4:**
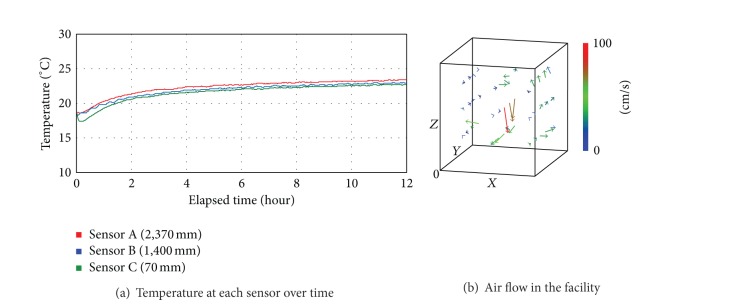
Temperature and air flow in the controlling condition of Case 2.

Figure 4 shows the actual measurements of the temperature and air flow in the facility in the controlling condition of Case 2 where LED lightings and air-flow fans were operating simultaneously. During the 12 hours, the average temperature in the facility gradually increased from 23.0°C to 24.0°C as shown in Figure 4(a). As for the average temperature at each sensor, that of sensor A on the top was 22.3°C, that of sensor B in the middle was 21.9°C, and that of sensor C at the bottom was 21.5°C, which indicated that the difference between the top and bottom was about 0.8°C, and thus the temperature distribution was relatively even. As for the air flow distribution in Figure 4(b), the range was from 0.075 m/s to 1.038 m/s, and the average air flow rate was 0.287 m/s, which indicated that the air flow became more active than in Case 1. Although this controlling condition might not be maintained depending on the atmospheric condition outside, it was confirmed that air-flow fans facilitated the air flow inside and reduced the difference between the top and bottom areas.

**Figure 5:**
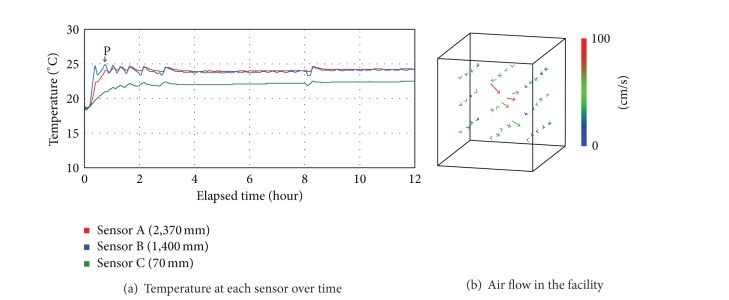
Temperature and air flow in the controlling condition of Case 3.

Figure 5 shows the actual measurements of the temperature and air flow, changing depending on the controlling condition of Case 3 where the LED lightings and cooling and heating devices were operating simultaneously. Point P at Figure 5(a) indicates the timing that the temperature reached the setting point (23°C) with the cooling and heating devices operating. The initial temperature was 18.6°C, and after 42 minutes from then on, the temperature stayed at 23°C on average. The average temperature at each sensor was 23.9°C (A), 23.9°C (B), and 22.0°C (C), which indicated that the difference was bigger than 1.9°C in Case 2. In addition, Figure 5(b) showed that in comparison with Case 2 whose range was from 0.063 m/s to 1.198 m/s, the maximum air flow rate was higher while the average air flow rate was low, down to 0.216 m/s. This was because a strong air flow was formed at certain areas with the cooling and heating devices operating but the air circulation was not smooth especially at corners. To maintain the temperature at a certain level in plant-growing facilities is of great importance when it comes to the environmental control of plant factories. Thus, the controlling condition of Case 3 where cooling and heating devices were operating must be reconsidered.

**Figure 6:**
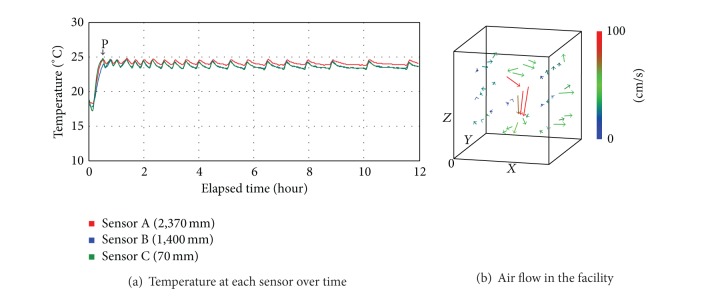
Temperature and air flow in the controlling condition of Case 4.

Figure 6 shows the actual measurements of the temperature and air flow, changing depending on the controlling condition of Case 4. In Case 4, LED lightings, cooling and heating devices, and air-flow fans were operating simultaneously. As shown in position P in Figure 6(a), the temperature drastically increased right from the beginning point of the experiment and reached the set point of 23°C within 23 minutes. The average temperature at sensor A was 24.0°C, that at sensor B was 23.6°C, and that at sensor C was 23.6°C, which indicated that the average temperature was 23.7°C and the difference between the top and bottom was 0.4°C. As stated earlier, cooling and heating devices were not operating in Cases 1 and 2, which involved the problem of maintaining the set temperature. These two cases should be analyzed in comparison with Case 3. As for temperature difference in comparison with Case 3, it decreased about 78.9% from 1.9°C to 0.4°C, which indicated that Case 4 was a more efficient controlling condition. In addition, the average air flow rate in the space was 0.354 m/s, which was 63.4% better than that of Case 3.  Figure 6(b) shows that circulation was better not only in certain areas with cooling and heating devices and air-flow fans installed, but also in general it was better. In other words, in the hybrid control condition where cooling and heating devices and air-flow fans were operating simultaneously, the difference in temperature between the top and bottom was minimized and the effect of indoor air circulation was more significant.

## 4. Conclusion

With a vertical multistage, closed-type plant factory was utilized as a testbed, the temperature and air flows changing in the space depending on the four different controlling conditions (see Table 2) were measured and compared in this study.  Table 3 shows the experimental results.

In Case 1 where only LED lightings were operating, both the average temperature and difference in temperature among different positions increased over time. The difference in temperature between the top and bottom was 2.7°C, the largest measurement of all. In contrast, Case 2, where air-flow fans were operating as well, showed that the difference in temperature between the top and bottom was as low as 0.8°C, 70.4% lower than that of Case 1. The average air flow rate was 0.287 m/s, which was 5 times better. In comparison with Case 3, where only cooling and heating devices were operating and Case 4 where both cooling and heating devices and air-flow fans were operating simultaneously, the difference in temperature between the top and bottom decreased about 78.9% from 1.9°C to 0.4°C in the controlling condition of Case 4. The time to reach the set point of temperature was reduced to about 45% from 42 minutes to 23 minutes. This indicated that the controlling condition of Case 4 was effective in reducing the running time of cooling and heating devices. The average air flow rate of Case 4 was 0.354 m/s, which was better in the others. In comparison to Case 3 where LED and cooling and heating devices were operating, the performance was 63.4% better. The experiment result indicated that the hybrid control of air flow devices such as cooling and heating devices and air-flow fans facilitated air flows in a facility and minimized the difference in temperature between the top and bottom areas. In the future, studies need to include simulations and actual measurements of the air flow, quantity, and arrangement of air-flow fans appropriate for the scale and structure of plant factories.

## Figures and Tables

**Table 1:** Specifications of the cooling/heating devices and air-flow fans.

Device	Cooling and heating devices	Air-flow fan
Model	S-W115AAW	SIV-200BC
Rated voltage	220 V	220 V
Frequency	60 Hz	60 Hz
Power consumption	1350 W	21 W
Area	37.5 m^2^	10 m^3^

**Table 2:** Device controlling conditions.

Case	Air conditioning	Fan	LED
Case 1	Off	Off	On
Case 2	Off	On	On
Case 3	On (23°C)	Off	On
Case 4	On (23°C)	On	On

**Table 3:** Temperature and air flow distributions depending on the controlling condition.

Case	1	2	3	4
Temperature [°C]				
Sensor A	23.9	22.3	23.9	24.0
Sensor B	23.6	21.9	23.9	23.6
Sensor C	21.2	21.5	22.0	23.6
Difference	**2.7**	**0.8**	**1.9**	**0.4**
Air flow [m/s]				
Minimum	0.026	0.075	0.063	0.107
Maximum	0.143	1.038	1.198	1.515
Average	**0.057**	**0.287**	**0.216**	**0.354**

## References

[1] Nam IU, Choi MD, Kim BJ (2012). Sustainable urban plants plant and air conditioning systems. *The Magazine of the Society of Air-Conditioning and Refrigeration Engineering of Korea*.

[2] Akamine T, Murase H, Murakami K Lighting environment control for plant factory optimization.

[3] Johkan M, Shoji K, Goto F, Hashida S-N, Yoshihara T (2010). Blue light-emitting diode light irradiation of seedlings improves seedling quality and growth after transplanting in red leaf lettuce. *HortScience*.

[4] Lim ST, Yang SR (2011). Is plant factory a sustainable alternative?. *Korean Journal of Agricultural Management and Policy*.

[5] Cho YY, Choi KY, Lee Y-B, Son JE (2012). Growth characteristics of sowthistle (Ixeris dentata Nakai) under different levels of light intensity, electrical conductivity of nutrient solution, and planting density in a plant factory. *Horticulture Environment and Biotechnology*.

[6] Hu MC, Chen YH, Huang LC (2014). A sustainable vegetable supply chain using plant factories in Taiwanese markets: a Nash-Cournot model. *International Journal of Production Economics*.

[7] Shimizu H, Saito Y, Nakashima H, Miyasaka J, Ohdoi K Light environment optimization for lettuce growth in plant factory.

[8] Mirabella O, Brischetto M (2011). A hybrid wired/wireless networking infrastructure for greenhouse management. *IEEE Transactions on Instrumentation and Measurement*.

[9] Kwon S-Y, Ryu S-H, Lim J-H (2013). Design and implementation of an integrated management system in a plant factory to save energy. *Cluster Computing*.

[10] Son JE (1993). Plant factory—a prospective urban agriculture. *Journal of Bio-Environment Control*.

[11] Kwon SY, Lim JH (2011). Improvement of energy efficiency in plant factories through the measurement of plant bioelectrical potential. *Informatics in Control, Automation and Robotics*.

[12] Hwang CH (1995). Temperature stress responses in plant and their agricultural application. *The Korean Journal of Breeding Science*.

[13] Farneti B, Schouten RE, Qian T, Dieleman JA, Tijskens LMM, Woltering EJ (2013). Greenhouse climate control affects postharvest tomato quality. *Postharvest Biology and Technology*.

[14] Park D-H, Park J-W (2011). Wireless sensor network-based greenhouse environment monitoring and automatic control system for dew condensation prevention. *Sensors*.

[15] Boo H-O, Heo B-G, Gorinstein S, Chon S-U (2011). Positive effects of temperature and growth conditions on enzymatic and antioxidant status in lettuce plants. *Plant Science*.

[16] Kang JH, KrishnaKumar S, Atulba SLS, Jeong BR, Hwang SJ (2013). Light intensity and photoperiod influence the growth and development of hydroponically grown leaf lettuce in a closed-type plant factory system. *Horticulture, Environment, and Biotechnology*.

[17] Hua J (2013). Modulation of plant immunity by light, circadian rhythm, and temperature. *Current Opinion in Plant Biology*.

[18] Choi JM, Park Y-J, Kang S-H (2014). Temperature distribution and performance of ground-coupled multi-heat pump systems for a greenhouse. *Renewable Energy*.

[19] Mathieu AS, Lutts S, Vandoorne B (2014). High temperatures limit plant growth but hasten flowering in root chicory (*Cichorium intybus*) independently of vernalisation. *Journal of Plant Physiology*.

[20] Yu IH, Cho MW, Lee SY, Chun H, Lee IB (2007). Effects of circulation fans on uniformity of meteorological factors in warm air heated greenhouse. *Journal of Bio-Environment Control*.

[21] Park MH, Lee YB (1999). Effects of CO_2_ concentration, light intensity and nutrient level on the growth of leaf lettuce in a plant factory. *Journal of the Korean Society for Horticultural Science*.

[22] Han JH (2004). *Development of a ventilation model for predicting mushroom house environment using 3-D CFD method [M.S. thesis]*.

[23] Fernandez JE, Bailey BJ (1994). The influence of fans on environmental conditions in greenhouses. *Journal of Agricultural Engineering Research*.

[24] Smith VC, Ennos AR (2003). The effects of air flow and stem flexure on the mechanical and hydraulic properties of the stems of sunflowers *Helianthus annuus* L.. *Journal of Experimental Botany*.

[25] Teitel M, Zhao Y, Barak M, Bar-lev E, Shmuel D (2004). Effect on energy use and greenhouse microclimate through fan motor control by variable frequency drives. *Energy Conversion and Management*.

[26] Teitel M, Levi A, Zhao Y, Barak M, Bar-lev E, Shmuel D (2008). Energy saving in agricultural buildings through fan motor control by variable frequency drives. *Energy and Buildings*.

[27] Liu G, Liu M (2008). Supply fan control methods for VAV systems using a fan airflow station. *ASHRAE Transactions*.

[28] Kitaya Y, Hirai H (2008). Effects of lighting and air movement on temperatures in reproductive organs of plants in a closed plant growth facility. *Advances in Space Research*.

[29] Thongbai P, Kozai T, Ohyama K (2010). CO_2_ and air circulation effects on photosynthesis and transpiration of tomato seedlings. *Scientia Horticulturae*.

[30] Andersson NE (2011). The influence of water stress and air velocity on growth of Impatiens walleriana and Petunia×hybrid. *Scientia Horticulturae*.

[31] López A, Valera DL, Molina-Aiz F (2011). Sonic anemometry to measure natural ventilation in greenhouses. *Sensors*.

[32] Coomans M, Allaerts K, Wittemans L, Pinxteren D (2013). Monitoring and energetic performance of two similar semi-closed greenhouse ventilation systems. *Energy Conversion and Management*.

